# Reward Circuitry Plasticity in Pain Perception and Modulation

**DOI:** 10.3389/fphar.2017.00790

**Published:** 2017-11-21

**Authors:** Marcos F. DosSantos, Brenda de Souza Moura, Alexandre F. DaSilva

**Affiliations:** ^1^Laboratório de Morfogênese Celular, Instituto de Ciências Biomédicas, Universidade Federal do Rio de Janeiro, Rio de Janeiro, Brazil; ^2^Programa de Pós-Graduação em Radiologia, Faculdade de Medicina, Universidade Federal do Rio de Janeiro, Rio de Janeiro, Brazil; ^3^Headache and Orofacial Pain Effort, Department of Biologic and Materials Sciences, School of Dentistry, Center for Human Growth and Development, Molecular and Behavioral Neuroscience Institute, University of Michigan, Ann Arbor, MI, United States

**Keywords:** chronic pain, reward circuitry, nucleus accumbens, prefrontal cortex, migraine

## Abstract

Although pain is a widely known phenomenon and an important clinical symptom that occurs in numerous diseases, its mechanisms are still barely understood. Owing to the scarce information concerning its pathophysiology, particularly what is involved in the transition from an acute state to a chronic condition, pain treatment is frequently unsatisfactory, therefore contributing to the amplification of the chronic pain burden. In fact, pain is an extremely complex experience that demands the recruitment of an intricate set of central nervous system components. This includes cortical and subcortical areas involved in interpretation of the general characteristics of noxious stimuli. It also comprises neural circuits that process the motivational-affective dimension of pain. Hence, the reward circuitry represents a vital element for pain experience and modulation. This review article focuses on the interpretation of the extensive data available connecting the major components of the reward circuitry to pain suffering, including the nucleus accumbens, ventral tegmental area, and the medial prefrontal cortex; with especial attention dedicated to the evaluation of neuroplastic changes affecting these structures found in chronic pain syndromes, such as migraine, trigeminal neuropathic pain, chronic back pain, and fibromyalgia.

## The Chronic Pain Burden

Pain is an experience that has been exhaustively investigated since the most remote civilizations. It is widely known as a clinical symptom shared by many pathological conditions. Pain is also the main factor that precipitates subjects to seek medical care (Fishman et al., 2010). When acute, nociceptive pain is driven by a primary medical condition, and directs the attention to a potential harmful stressor that must be opposed (Elman and Borsook, 2016).

Beyond nociception, pain is an uncreditable complicated phenomenon that comprises sensory/discriminative, affective/motivational and cognitive/evaluative dimensions (Melzack and Casey, 1968). Moreover, it often progresses from an acute benign stage to a chronic, debilitating, and in some instances, unbearable status. In this context, chronic post-surgical pain is extremely relevant, since its occurrence cannot be predicted by the kind of surgical procedure performed (Lavand’homme, 2017). Furthermore, it has been estimated that this type of condition occurs in 1 of every 10 surgical procedures and it becomes an unbearable condition in 1 of every 100 surgeries (Breivik and Stubhaug, 2008). Conversely, other chronic pain conditions may unexpectedly evolve from an initial injury, without a history of surgery (e.g., complex regional syndrome) or initiate as the chief complaint (e.g., trigeminal neuralgia) (Mao, 2017). Chronic pain syndrome is a health issue that afflicts a large fraction of the population (Elliott et al., 2002; Patel et al., 2012). It represents an immense burden for the individuals affected, and produces considerable socio-economic impact due to the high costs with health care systems, absenteeism and reduced productivity (Patel et al., 2012).

Epidemiological studies have estimated that currently, roughly 100 million Americans experience pain, leading to a cost of approximately 600 billion dollars every year (Nahin, 2015). On the other hand, chronic pain is present in approximately 25% of the general population or 25 millions of Americans, being portrayed as an epidemic urgent medical condition of our modern society (O’Connor, 2009; Nahin, 2015). Noteworthy, acute and chronic pain should not be differentiated merely based on an arbitrary chronological cut-off point. Another important fact that must be highlighted is that chronic pain is a collective term rather than a nosological entity, considering that heterogeneous symptomatic profiles can differentiate nociceptive (e.g., osteoarthritis and low back pain) from neuropathic pains (e.g., trigeminal and post-herpetic neuralgias).

## Chronic Pains Pathophysiology: An Unsolved Puzzle

Notwithstanding the efforts and the advances in the route to delineate the biological factors that ultimately lead to chronic pain, the cascade of events that result in pain chronification are yet considered part of an enigmatic puzzle. The still scarce information regarding this whole process probably helps to understand the struggle reported by clinicians in the chronic pain management (Kheshti et al., 2016). Chronic pain is a phenomenon with multifactorial, highly complex and usually poorly understood etiologies. It is also characterized by marked individual differences (Cohen and Mao, 2014). Therefore, proper pain management should include multimodal and customized therapies (Sarzi-Puttini et al., 2012). Chronic pain can be an initial complaint but it in some cases it evolves from an acute condition. When this is the case, expectations are inheritably affected, resulting in additional suffering (Mao, 2017). The development of side effects, tolerance or dependence, especially with opioid analgesics can prevent the long-term use of several drugs used for chronic pain management, an aspect that must also be considered (Trang et al., 2015). Physicians and all other health care providers must be fully prepared to deal with these challenges, with the purpose of providing appropriate pain control (Breivik et al., 2013; Johnson et al., 2013).

The lack of specific biomarkers and inconclusive results of diagnostic exams are additional challenges in the diagnosis and treatment of most the chronic pain conditions (Mao, 2012). Another crucial aspect that must be considered is that chronic pain and neuropsychiatric conditions are significantly comorbid (Tasmuth et al., 1996; Jess et al., 1998; Linton, 2000; Dominick et al., 2012). The results of a clinical study indicates that pain, especially when affecting multiple locations is a risk factor for depressive and anxiety disorders (Gerrits et al., 2014). Anxiety and depression could play a role in the persistence of chronic pain and therefore influence the efficacy of chronic pain management (Currie and Wang, 2005; Dahan et al., 2014). Pain also produces negative impacts on sleep, social and work functioning with a direct effect on the quality of life. Not surprisingly, chronic pain patients are hospitalized much more often than the general population (Collett, 2011).

The pathophysiology underlying chronic pain have been extensively studied in the past few years and distinct mechanisms, ranging from maladaptative neuroplastic alterations (Apkarian et al., 2004; DaSilva et al., 2007a,b, 2008), central and peripheral sensitization (Ultenius et al., 2006; Smith et al., 2008; Basbaum et al., 2009) and epigenetics (Imai et al., 2011; Zhang et al., 2011; Buchheit et al., 2012; Tajerian et al., 2013; Descalzi et al., 2015) have been suggested. More recently, the role of glial cells, especially microglia, has been acknowledged (Ohara et al., 2009; Takeda et al., 2011; Katagiri et al., 2012; Ji et al., 2013; Tenorio et al., 2013; Cahill and Taylor, 2017). While the mechanisms underlying chronic pain have been at least partially identified, much less is known about the circuits responsible for the transition from acute to chronic pain. Besides, the individual susceptibility to the development of chronic pain is still poorly comprehended. In this scenario, it has been hypothesized that a differential engagement of reward neural circuits might play a significant role (Cahill et al., 2014; Baliki and Apkarian, 2015; Borsook et al., 2016; Mitsi and Zachariou, 2016).

## Pain Neuroanatomy

**FIGURE 1:**
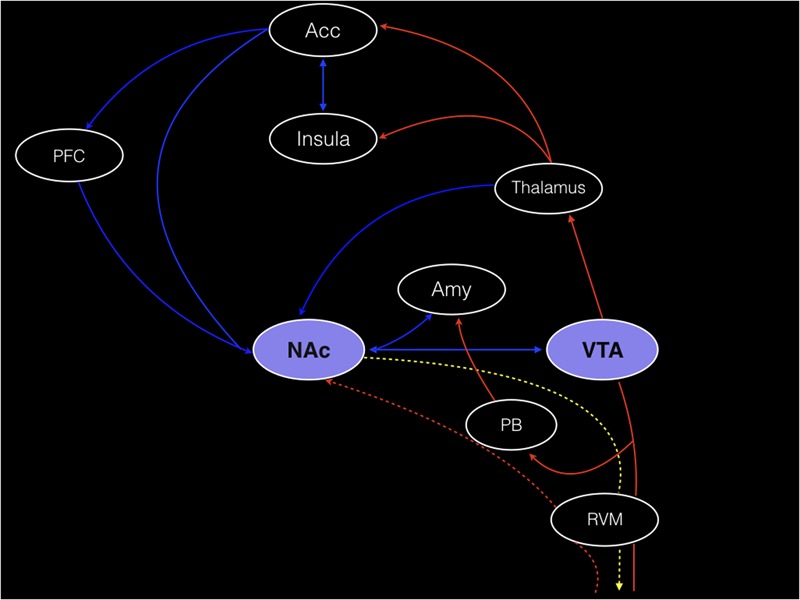
Corticolimbic circuits and the main components of the reward circuitry are illustrated. Red lines: the nucleus accumbens (NAc) receives afferent nociceptive information mainly via connections with the anterior cingulate cortex (ACC), amygdala (Amy), thalamus, and parabrachial nucleus (PB). Possible direct connections from the spinal cord to the NAc (red dotted line) are also shown. Blue lines: corticostriatal projections arising from the prefrontal cortex (PFC) and ACC. The connections between the Amy, thalamus and ventral tegmental area (VTA) and the NAc are also represented by blue lines. The descending pathway from the NAc to the spinal cord, that putatively regulates nociceptive information, probably through the rostral ventromedial medulla (RVM), is represented by a gold dotted line. Adapted by permission from Macmillan Publishers Ltd: *Nature Neuroscience* (Navratilova and Porreca, 2014), copyright 2014.

The broad network of nervous system structures related pain, which has been recently incorporated in the pain connectome (Kucyi and Davis, 2015), is embodied by a set of both cortical and subcortical brain regions. In this system, a prominent contribution might be attributed to the primary and secondary somatosensory cortex (S1 and S2, respectively), which along with the insula, encode the basic aspects of the noxious stimuli (e.g., pain intensity, quality, location, and duration); and to the prefrontal cortex (PFC), that cooperating with the anterior cingulate cortex (ACC), amygdala, insula, VTA, NAc, lateral and medial habenula (MHb and lHb) processes the affective features of pain. Nonetheless, those structures also act on more specific functions. ACC seems to be associated with unpleasantness sensations and fear avoidance while the amygdala is a region classically linked to fear. Among the structures described, those related to the mesolimbic system (e.g., VTA and NAc) and particularly the VTA projections and dopaminergic neurotransmissions in the NAc have been largely associated with reward/motivation (**Figure 1**). Nevertheless, the reward circuitry is not restricted to these areas. Instead, a natural reward is additionally processed by other brain regions, including the PFC and the ACC (Navratilova et al., 2015). More specifically, it has been described that the integration of the evasiveness driven by pain occurs partially in the rostral ACC (Tracey and Mantyh, 2007; Becerra et al., 2013). Functional magnetic resonance imaging (fMRI) and positron emission tomography (PET) studies have demonstrated functional activity (Wanigasekera et al., 2012) and dopaminergic activation (Scott et al., 2006) induced by noxious stimuli in some of those areas (e.g., NAc and VTA). Furthermore, the dopaminergic (D2 receptor) activation induced by noxious stimulus was positively correlated with individual variations in the ratings of both sensory and affective dimensions of the pain experience (Scott et al., 2006). Anatomical and functional reorganization of the reward circuitry structures have been reported with chronic pain (Apkarian et al., 2011).

Other important brain areas for pain are the hypothalamus and parabrachial nucleus, functionally connected to the autonomous nervous system; the thalamus, which relays ascending nociceptive pathways to the somatosensory cortex; the cerebellum and the anatomical components of the reticular formation, such as the periaqueductal gray (PAG), the rostral ventromedial medulla (RVM) and the locus coeruleus (LC) (Willis and Westlund, 1997; Ossipov et al., 2010, 2014; Lamm et al., 2011; Benarroch, 2016).

The axis (PAG-RVM-spinal dorsal horn) is interpreted as the central structure in the descending pain modulatory system (Mason, 2005). It is broadly accepted that both PAG and RVM stimulation produce antinociceptive effects through descending serotonergic neurons, therefore acting at through serotonin (5-HT) receptors (Aimone et al., 1987). More important, the analgesic effects of several opioids, antidepressants, and non-steroidal anti-inflammatory drugs greatly depend on the integrity of all these structures (Ossipov et al., 2014). Additionally, the participation of endocannabinoid receptors CB1 has also been suggested (Dogrul et al., 2012). The functions of the major pain-related brain structures discussed in this manuscript are summarized in the **Table 1**.

**Table 1:** An overview of the main brain structures related to pain discussed in this article.

Brain region	Function in pain
Amygdala	Related to the affective-motivational dimension of pain (Neugebauer et al., 2004) as well as the modulation of nociceptive stimuli (Neugebauer, 2015). Clinical pain produces peaks of activation in the laterobasal amygdala while experimental pain is often associated with an increased signal within its superficial region (Simons et al., 2014).
Anterior cingulate cortex (ACC)	Important for cognitive and affective reactions to pain (Tracey and Mantyh, 2007; Becerra et al., 2013). Increased regional blood flow driven by pain has been mostly reported in the mid-cingulate and perigenual regions (Peyron et al., 2000). It has been suggested that the activity of the perigenual ACC would be linked to the affective reaction triggered by pain unpleasantness, while the mid-cingulate part would be related to the cognitive features (motor inhibition and response selection) of pain (Vogt et al., 1996). However, the differential pain-related activation of these two areas of the ACC has been discussed (Peyron et al., 2000).
Habenula (Hb)	Important component of the reward circuitry. This small subcortical structure regulates anxiety, pain and stress and inhibits reward under such conditions (Sartorius et al., 2010; Elman and Borsook, 2016). It promotes an inhibitory regulation to the NAc dopaminergic neurons and to the mPFC neurons (Lee and Goto, 2011). Therefore, it regulates the dopamine levels in striatum. Hb also modulates the release of other neurotransmitters related to aversive behaviors through its connections to the brainstem and basal forebrain related structures (e.g., norepinephrine-locus coeruleus; raphe nuclei-serotonin; acetylcholine-nucleus basalis of Meynert) (Elman and Borsook, 2016).
Insula	Insula activation has been consistently found in fMRI pain studies (Apkarian et al., 2005). A somatotopic representation of painful thermal stimuli has been demonstrated in the dorsal posterior insula (Brooks et al., 2005). In addition, rostral anterior insula activation has been connected to clinical pain while caudal anterior insula activity has been associated with experimental pain (Schweinhardt et al., 2006).
Nucleus accumbens (NAc)	A prominent component of the reward circuitry. A decrease in the blood oxygenated level dependent (BOLD) signal at the onset (aversive) and an increase at the offset (rewarding) of painful heat stimulus has been found in the NAc of healthy subjects (Becerra and Borsook, 2008). NAc activity in response to the offset of painful stimuli allowed the differentiation between chronic low back pain and healthy subjects at very high accuracy (Baliki et al., 2010).
Prefrontal cortex (PFC)	Involved in the cognitive-attentional aspects of pain (Peyron et al., 2000). Dorsolateral prefrontal (DLPFC) has been implicated in both pain suppression and detection (Seminowicz and Moayedi, 2017). Structural changes in the DLPFC has been demonstrated in chronic pain (Apkarian et al., 2004).
Primary (S1) and secondary (S2) somatosensory cortex	Encode the basic aspects of noxious stimuli (e.g., pain intensity, quality, location, and duration) (Peyron et al., 2000).
Reticular formation	Supraspinal control of the nociceptive transmission at the level of the laminae I, II, and V of the dorsal horn of the spinal cord. Its main components are the periaqueductal gray (PAG), the rostral ventromedial medulla (RVM), and the locus coeruleus (LC). Such regions integrate the descending pain modulatory system (Ossipov et al., 2014).
Thalamus	Primarily related to the sensory-discriminative aspects of pain. Bilateral activation during painful stimulus possibly reflects its involvement in attentional networks (Peyron et al., 2000).

## Pain and Mental Comorbidities

It is well-known that chronic pain is comorbid with depression (Mayor, 2016). In addition, anxiety disorders, schizophrenia and bipolar disorder, are also comorbid with pain (Nicholson and Verma, 2004). For example, a recent study has revealed that chronic back pain (CBP) is related to higher odds of stress, anxiety, psychosis, sleep disturbances and depression. Interestingly, the predictive value of the catastrophizing pre-surgical scores to the development of chronic post-surgical pain has also confirmed in patients that undertaken spine surgeries (Havakeshian and Mannion, 2013). In fact, it has been advocated that the psychosocial and functional consequences of chronic pain determine the pain experience and should be contemplated in chronic pain classifications (Turk et al., 2016). Noteworthy, the risk of suicide is considerably high among chronic pain patients (Juurlink et al., 2004; Hassett et al., 2014). It has been admitted that the proclivity for suicide observed in such patients, may be influenced by changes in reward and anti-reward circuitries (Elman et al., 2013).

Taken collectively, these data sustain the concept of the reward circuitry participation in chronic pain and related comorbidities. In addition, the reward circuitry is probably involved in the transition from acute to chronic pain, which might occur through a functional rearrangement of its main components (L’vov et al., 1989). Furthermore, comorbidity exists between persistent pain and diseases that are accompanied by altered dopaminergic function such as major depression, drug addiction (Jarcho et al., 2012) and Parkinson’s disease (PD) (Skogar and Lokk, 2016; Young Blood et al., 2016). In those conditions, chronic pain correlates to structural and functional changes in pain-related areas, such as PFC, cingulate cortex (CC), and insula. In addition, a study that assessed pain in PD on and off levodopa, reported higher pain ratings measured by a visual analog scale (VAS) during the off period (Nebe and Ebersbach, 2009).

Altered dopamine function has been reported in major depression (Laasonen-Balk et al., 1999; Corrigan et al., 2000). However, the specific mechanism that explain this relationship are not fully understood. Data from photon emission computerized tomography (SPECT) evaluating the uptake of a high-affinity dopamine transporter specific radioligand in the basal ganglia of depressed patients and healthy controls, suggested that the primary dysfunction of the dopaminergic system in depression is related to the up-regulation of dopamine transporter density, causing a more effective re-uptake of dopamine into the presynaptic neurons, thus resulting in a lower concentration of dopamine in the synaptic gap and a consequent decrease in the dopaminergic neurotransmission (Laasonen-Balk et al., 1999). Also, supporting the presence of the link between dopamine dysfunction and depression, pramipexole, a D2 receptor agonist improved depressive symptoms in patients with major depression, when administrated at doses of 1 mg per day and even more pronouncedly when administrated at doses of 5 mg per day (Corrigan et al., 2000). Another possible mechanism that could explain the association between depression and dopamine dysfunction, involves psychological stress, which is considered a general but basic symptom of depression. In effect, stress activates dopamine neurons in the ventral tegmental area (VTA) and results in a tonic dopamine release in the nucleus accumbens (NAc) (Horvitz, 2002). Stress also stimulates the function of the cAMP response element binding (CREB) in the NAc (Barrot et al., 2002) and its activity is mediated by dopamine receptors (Yan et al., 1999; Dudman et al., 2003). Such information supports the connection between depression and dopamine function (Finan and Smith, 2013). Moreover, hyperalgesia can be caused by chronic stress, possibly due to changes in the NAc dopaminergic function (Quintero et al., 2000; Wood, 2004), leading to the hypothesis that the changes found in the VTA dopaminergic neurotransmission induced by prolonged stress exposure, would be related to allostatic adaptations to chronic pain, which in turn would trigger and/or contribute to maintain anxiety and depression (Massaly et al., 2016). Substantiating the presence of overlapping mechanisms related to chronic pain and depression or other psychiatric disorders that involve an altered dopaminergic function, a recent PET study, found differences in the availability of dopamine D2/D3 receptors in the striatal regions (e.g., caudate nucleus, putamen, and NAc) of fibromyalgia patients with and without depression (Ledermann et al., 2016).

## An Overview of the Reward System Elements

Among the pain-related brain structures previously discussed, the NAc, the PFC and the VTA are the classical components of the brain reward circuitry (Goeders and Smith, 1983). Completing the reward/motivational circuitry are other important areas, including the ACC and the lateral orbitofrontal cortex (OFC) (Navratilova et al., 2015). These structures are illustrated in the **Figure 1** and their functions are summarized in the **Table 1**. In this circuitry, dopamine midbrain neurons, especially those connecting the VTA to the NAc and the NAc shell to the striatum (striato-nigro-striatal pathway) are considered the key elements (Elman and Borsook, 2016). Not surprisingly, dysfunction of the VTA-NAc mesolimbic pathway has been associated with depression (Russo and Nestler, 2013; Su et al., 2016) and neuropathic pain (Ozaki et al., 2002; Taylor et al., 2015). Remarkably, previous studies have evidenced a dual behavior of those dopaminergic neurons, since a phasic burst occurs in response to an unexpected reward while a phasic firing inhibition occurs when an expected reward is not received (Fields et al., 2007; Schultz, 2007).

Another important feature of the reward circuitry is that valence and salience of environmental stimuli are handled by distinct subgroups of midbrain neurons. In this regard, most of those neurons encode valence, increasing their firing in response to rewarding and decreasing their firing in response to aversive stimuli. Conversely, a second class of neurons react to both aversive and rewarding stimuli and therefore encode salience (Schultz, 2013).

Moreover, the prominent dopaminergic projections, mostly deriving from the VTA, have opposite actions in the NAc medium spiny neurons, depending on the targeted dopamine receptor and the associated pathway. When acting at the direct pathway, via the interaction with D1 receptors, dopamine stimulates medium spiny neurons and promotes positive affect. On the contrary, when acting at the indirect pathway, via D2 receptors, dopamine inhibits medium spiny neurons and a negative affect evolves (Gerfen and Surmeier, 2011).

The activity of the VTA-NAc circuit is modulated by the signals related to different types of neuromodulators [e.g., glutamate, γ-aminobutyric acid (GABA), serotonin, dopamine, and orexin]. Such signals originate in both cortical and subcortical structures that participate in mood, memory, emotions, stress and pain, such as the amygdala, the medial PFC and the Hb (Hikosaka et al., 2008). Some of the regulatory pathways are: the excitatory orexinergic projections from the hypothalamus (mainly lateral hypothalamus) to the VTA; the excitatory glutamatergic projections from the hippocampus to the NAc and from mPFC and stria terminalis to the VTA and the inhibitory GABAergic neurons extending from the NAc to the VTA (Kalant, 2010).

Although anatomically small, the Hb (the latin term for “little rein”), is another chief element of the reward circuitry. The Hb controls the dopamine levels in the striatum. Furthermore, the connections between the Hb and raphe nuclei are especially important since the serotonergic system plays a role in both depression and pain (Stamford, 1995; Gao et al., 2008). In animal models, lesions in the LHb produces a reduction in depressive-like behaviors by elevating the levels of serotonin in the dorsal raphe nucleus (Yang et al., 2008), which strongly suggests the participation of the LHb in the control of pain-related depression (Li et al., 2017). Supporting this notion, an increase in the LHb activity along with a reduced activity in the dorsal raphe nucleus has been found in an experimental model of neuropathic pain. This model has also resulted in pain and depressive-like behaviors, which have been reversed by LHb lesions (Li et al., 2017).

Interestingly, while the LHb modulates serotonin and norepinephrine releases, the acetylcholine neurotransmission is under the MHb regulation (Velasquez et al., 2014). The effects of electrical stimulation of the habenula have been examined and the results indicates that this procedure results in analgesia in the formalin test (Cohen and Melzack, 1986). Moreover, such effect is reversed by a direct injection of naloxone into the habenula (Wagner et al., 1977). From a clinical/neurosurgical perspective, bilateral stimulation of the LHb afferent bundles (e.g., stria medullaris thalami) has been also successfully applied to treat major depression in a patient that had been refractory to conventional therapies for 9 years (Sartorius et al., 2010). Nonetheless, the effects bilateral stimulation of LHb on pain, especially in cases of patients with significant psychopathology, must be further explored in depth. Remarkably, the presence of specialized projections helps to explain the opposite functions of the LHb and the laterodorsal tegmentum. While the LHb neurons synapse on the VTA dopaminergic neurons that project to the mPFC and to the rostromedial tegmental nucleus, the laterodorsal tegmentum neurons synapse on VTA dopaminergic neurons that project to the NAc lateral shell (Lammel et al., 2012). Consequently, activation of VTA neurons from the LHb projections results in aversion. On the other hand, activation of VTA inputs from the laterodorsal tegmentum produces reward (Lammel et al., 2012).

Opioid neurotransmission is an additional reward system constituent and it is directly associated with the reward experience caused by pain relief (Navratilova et al., 2015). It is composed by a diffuse network that encompasses the PAG, the NAc and the VTA though not restricted to them. Opioids act through interaction with a super-family of G-protein-coupled receptors, namely μ, κ and δ, expressed both in the central nervous system (CNS) as well as in the peripheral nervous system (PNS) (Trang et al., 2015). The activation of μ-opioid receptors facilitate dopamine release and mediates pleasure, while the activation of κ-opioid receptors mediate aversion (e.g., irritability, dysphoria, and pain) (Wise and Koob, 2014). An interaction between the opioidergic systems has been documented, where enhanced function of κ-opoid neurotransmission in the NAc suppressed the effects of μ-opioid receptor agonist morphine (e.g., reward and facilitation of the dopaminergic neurotransmission in mesolimbic neurons), in a model of inflammatory pain induced by formalin injection (Narita et al., 2005). According to the authors, those results would highly support the use of morphine for inflammatory pain. ACC is another important region to the opioidergic system, since it contains neurons that express high levels of opioid neuropeptides and opioid receptors (Vogt, 2005). Painful stimulus promotes release of endogenous opioids in the ACC which is negatively correlated with pain affective scores (Zubieta et al., 2001). Such findings suggest that the ACC opioid components are related to the affective instead of the sensory dimension of pain (Lee et al., 2014; Navratilova et al., 2015).

## Plastic Changes in the Reward System Related to Chronic Pain

During the course of a transient noxious stimulus, its onset is aversive, acting as a punisher, while its offset represents a potential reward, thus differentiating an early (aversive) and a late (appetitive) element (Baliki et al., 2010). In sum, pain is considered a punisher, whereas its relive proportionate a negative reinforcement. In fact, the introduction of a noxious stimulus, prompts a decision, which involves an estimation of the anticipated pain associated with a prediction of costs and utility, prior to a proper behavioral response, in the light of other competing goals (Baliki et al., 2010). Fear of pain is one of the most witnessed phenomenon in chronic pain patients. It produces a selective attention, resulting in a hypervigilance to potential painful stimuli. Ultimately, this common chronic pain feature translates the aforementioned concepts to the clinical setting (Keogh et al., 2001).

### Evidence from fMRI Studies

There is mounting scientific evidence showing the occurrence of a reward circuitry plasticity in different chronic pain syndromes, with an especial attention to the process that guides the transition from acute or episodic pain, to chronic pain. In this regard, NAc has emerged as a crucial structure. One research study revealed that the transition from a subacute to a chronic state of low back pain at 1-year follow-up, can be predicted by an increased connectivity between the ventromedial PFC and the NAc (Baliki et al., 2012). Compatibly, in a groundbreaking neuroimaging study exploring the CNS effects of acute thermal stimuli, Baliki et al. (2010) found that the NAc activity permitted a differentiation, at very high accuracy, between CBP patients and healthy controls, with deactivation in patients and phasic activation in healthy subjects during the offset of the painful stimulus. Furthermore, those changes were correlated to the oscillations in the functional connectivity between the NAc and other brain regions. In CBP patients NAc activity was positively correlated to the MagINS namely the part of the insula that is related to the magnitude of thermal pain perception in healthy subjects (Baliki et al., 2009), but not to the PFC activity, an association that was inverted in CBP patients (Baliki et al., 2010). That study provided novel and promising information regarding the fundamental role of the NAc to the development of chronic pain, particularly CBP. Although the methodology of that study did not focus on the clinical evaluation of the CBP patients enrolled, the results obtained are supported by the clinical features observed in studies involving CBP patients. It has also been supported that the increased activity that takes place in NAc associated with pain relief (e.g., offset of a thermal stimulus in healthy subjects) (Becerra and Borsook, 2008; Baliki et al., 2010) or relief from expected pain (Leknes et al., 2011), reflects the reward prediction error, which has been classically linked to the mesolimbic dopaminergic neurons (Navratilova et al., 2015). In other words, phasic activity of the midbrain dopaminergic neurons would signal an incongruity between actual and predicted reward (Colombo, 2014).

Chronic back pain is a condition highly associated with anxiety and depression (Nicholas et al., 2011). Corroborating these clinical observations, a concise psychiatric evaluation, conducted through the CBP Brief Scale for Psychiatric problems in Orthopedic Patients (BS-POP), a simple instrument that measures the presence of psychiatric problems and the quality of life in orthopedic patients (Yoshida et al., 2011), showed a reduction in the NAc activity of subjects that exhibited higher BS-POP scores (Kaneko et al., 2016). The same individuals had increased pain and decreased quality of life. Furthermore, the results of experimental chronic pain models suggest that these changes could be driven by variations in the dopaminergic, opioidergic, and/or glutamatergic signaling within the NAc (Goffer et al., 2013; Terzi et al., 2014). Therefore, the NAc activity might be an indicator of motivational/emotional abnormalities in CBP patients and possibly a reliable predictor of the treatment success. For instance, a dysfunctional NAc might predict negative results for surgical interventions in CBP patients (Kaneko et al., 2016). However, this hypothesis lacks confirmation. Furthermore, the specific mechanisms involved (opioidergic, serotonergic, dopaminergic, glutamatergic) must be dissected.

Changes in the reward circuitry, including gray matter atrophy in the PFC, insula and NAc have also been shown in CRPS (complex regional pain syndrome), another painful disorder characterized by a challenging diagnosis, clinical management and still unclear pathophysiology (Geha et al., 2008). Similar findings have also been found in neurodegenerative disorders that comorbid with pain, such as PD and multiple sclerosis (MS), which can serve as models to study the impact of reward circuitry plasticity in chronic pain (Polli et al., 2016; Seixas et al., 2016).

Among the non-motor symptoms in PD, pain has a high prevalence (Broen et al., 2012). Conversely, it is usually a neglected and undertreated symptom. Accordingly, there is limited scientific evidence regarding the mechanisms of pain in PD (Zis et al., 2015). It has been revealed that in PD, chronic pain correlates to structural and functional changes in pain-related areas, such as the DLPFC, CC, and insula. Strikingly, the same study found a disconnection between the NAc and the left hippocampus in the cohort analyzed (Polli et al., 2016). Considering the eminent role of the NAc in chronic (Baliki et al., 2010) and neuropathic pain (DosSantos et al., 2012b; Chang et al., 2014) and the relevance of its intrinsic connections with the hippocampus (Kahn and Shohamy, 2013), such results might indicate that a neuropathic mechanism might underlie the chronic pain of PD patients (Polli et al., 2016).

Neuropathic pain, with a high risk of chronification is also a recurrent symptom in MS, a demyelinating condition well-known for its deleterious effects in the somatosensory system (Osterberg et al., 2005). Nevertheless, structural and functional changes in areas linked to reward processing, especially motivation, have been shown in MS patients with chronic pain. The areas exhibiting differential activation pattern and/or cortical thickness were the caudate nucleus, the NAc and the temporal lobe (Seixas et al., 2016). Although interesting, these finds must be taken cautiously and the relationship between chronic pain in MS and altered reward circuitry functioning must be further explored in depth, considering that an impaired decision-making, related to the evaluation of reward/punishment and possibly reflecting a compromised emotional reactivity but not dependent on pain, has been found in MS (Kleeberg et al., 2004).

### Evidence from PET Studies

**FIGURE 2:**
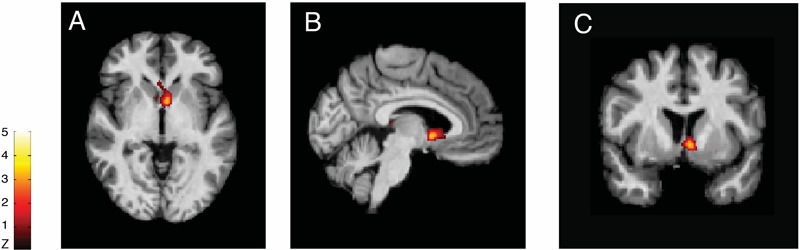
Reduced μ-opioid receptor binding potential (μOR BPND) in Trigeminal Neuropathic Pain. Decreased μOR BPND in the left NAc in axial **(A)**, sagittal **(B)**, and coronal **(C)** planes (T = 3.2) (DosSantos et al., 2012b).

**FIGURE 3:**
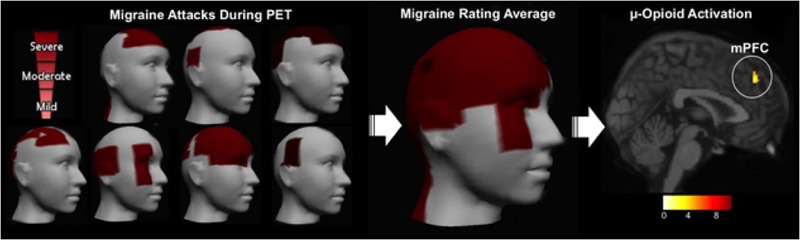
Migraine severity and ictal μ-opioid activation. **(Left)** Pain intensity and location in each migraine patient evaluated. **(Center)** 3D image representing the average rating of both the pain intensity and the pain location. **(Right)** Decreased μOR BPND in the mPFC during the migraine ictal phase as compared to the interictal phase (DaSilva et al., 2014).

Using PET, with the selective radioligand [11C]carfentanil, our group demonstrated activation of the the μ-opioid neurotransmission (represented by a decreased availability of μ-opioid receptors), *in vivo*, precisely in the NAc of trigeminal neuropathic pain patients (DosSantos et al., 2012b) (**Figure 2**). Similar changes in the reward circuitry had been observed before in episodic migraine patients, during the course of spontaneous migraine headache attacks (DaSilva et al., 2014). However, in that case, instead of the NAc, the μ-opioid activation, represented by a decreased availability of μ-opioid receptors was found in the mPFC (**Figure 3**), suggesting the possible contribution of this area to the migraine pathophysiology (Zhang et al., 2016). The results of the two aforementioned studies also denote that specific regions within the reward system could be active by different chronic pain syndromes, though many other limbic regions that not the PFC (e.g., insula and amygdala) have been related to migraine (Stankewitz and May, 2011). Furthermore, a previous study with fibromyalgia patients described a significant correlation between the changes in the striatum μ-neurotransmission and the affective pain dimension, assessed through the McGill pain questionnaire. An important difference is that in the fibromyalgia study, changes were widespread through pain-related structures, including areas that play a significant role in the emotional aspects of pain, such as the CC and the amygdala (Harris et al., 2007).

It is very likely that the dysregulated opioidergic neurotransmission described might impact the effects of analgesic opioid drugs in trigeminal neuropathic pain, fibromyalgia, migraine, and other painful syndromes. In addition, there is evidence that inflammatory pain can lead to a desensitization of μ-opioid receptors in the VTA, which turns increases heroin self-administration (Hipólito et al., 2015). Such findings clearly indicate that a loss of μ-opioid receptor function in the mesolimbic dopamine pathway may decisively participate in the escalation of opioid doses that may ultimately lead to opioid abuse. The presence of a compartment specificity (e.g., cell body and nerve terminals) in the VTA μ-opioid receptor desensitization has also been revealed (Hipólito et al., 2015). However, the significance of those findings must be further explored.

**FIGURE 4:**
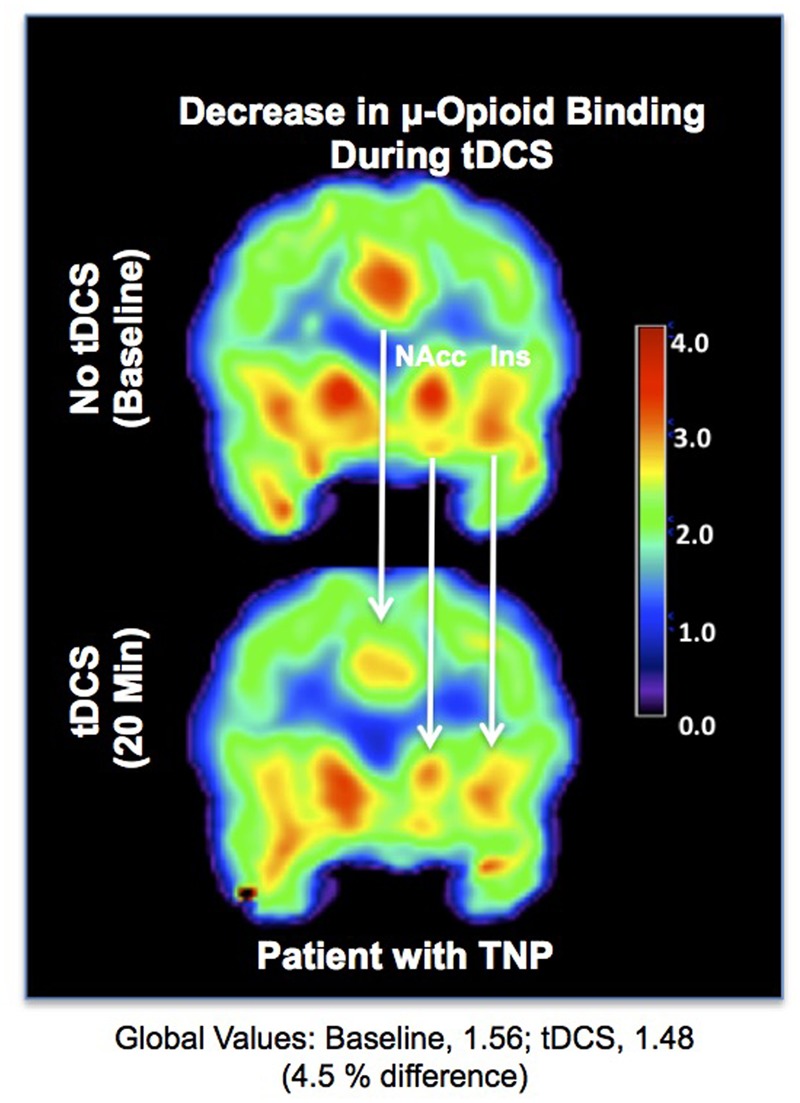
Reduced μOR BPND related to transcranial direct current stimulation (tDCS). **(Upper)** μOR BPND during the baseline. **(Lower)** μOR BPND during anodal motor cortex tDCS in the ACC, NAc, and insula (DosSantos et al., 2012a).

The functional evaluation of the opioidergic system *in vivo* has also helped to elucidate the mechanisms of pain relief generated by novel methods of non-invasive neuromodulation. Illustrating this aspect, in a previous study, we were able to demonstrate activation of μ-neurotransmission, possibly reflecting an increase in the endogenous opioid release in the ACC, NAc and insula, during anodal transcranial direct current stimulation (tDCS) of the motor cortex contralateral to the painful side, in a patient with post-herpetic neuralgia (DosSantos et al., 2012a) (**Figure 4**). Thus, it is possible that ingrained neuroplastic changes in the reward circuitry could be reverted by neuromodulatory techniques.

Regarding the evaluation of the dopaminergic system in painful syndromes, a significant increase in the BP_ND_ of the D2/D3 dopamine receptors selective radioligand [11C] raclopride has been found within the striatum of migraine patients during both the spontaneous headache attack and during allodynia induced by a sustained thermal pain threshold challenge, when compared to the non-headache (interictal) period (DaSilva et al., 2017). A decreased dopamine release (e.g., change in the [11C] raclopride BP_ND_ from baseline to the activated state) and thus lower D2/D3 receptor activation has also been reported in the ventral striatum of chronic non-neuropathic back pain patients, when compared to controls, during a pain challenge (Martikainen et al., 2015). Those results, contribute to sustain the concept that an altered dopaminergic function within the reward circuitry plays an important role in the pathophysiology of different painful conditions, including migraine and back pain. Indeed, altered striatal dopaminergic neurotransmission had been previously found in PET studies involving patients with other painful disorders, such as atypical facial pain (Hagelberg et al., 2003a) and burning mouth syndrome (Hagelberg et al., 2003b).

## Molecular Targets at the Reward Circuitry for Pain Relief

As previously discussed, reward circuitry components participate in depression and chronic pain mechanisms. Hence, they are potential targets for analgesics and antidepressant drugs. However, the specific molecular targets for chronic pain treatment within this circuitry remain partially unexplored. Early experimental studies have revealed that the dopamine release in the NAc induced by rewarding drugs, such as morphine and cocaine, can be suppressed by neuropathic pain (Ozaki et al., 2002; Taylor et al., 2015). Moreover, it has been reported that the pain relief produced by an intrathecal injection of pregabalin in a model of spinal nerve ligation is accompanied by increase in the levels of NAc dopamine, and effect detected during the early but not during the late phase of the experimentally induced neuropathic pain (Kato et al., 2016). Conversely, intra-accumbal infusions of D1- (SCH-23390) and D2-like (Sulpiride) dopamine receptors antagonists reduces stress-induced analgesia in a dose-dependent manner, in the early and late phases of an experimental model of persistent inflammatory pain, though with more pronounced effects found throughout the late phase (Faramarzi et al., 2016).

Recent studies have found changes in the reward circuitry related to abusive use of anabolic androgenic steroids (AASs). In a previous study, chronic use of nandrolone resulted in anhedonic behavior alongside changes in the noradrenergic, dopaminergic, and serotonergic neurotransmissions within the NAc of rats (Zotti et al., 2013). In another study, nandrolone induced conditioned place preference (CPP) which was accompanied by a decreased expression of D1 dopamine receptors in the NAc of adult mice (Martínez-Rivera et al., 2015). Furthermore, chronic pretreatment of with nandrolone has been proved to increase the reward related to low-dose morphine, which could contribute to an increased susceptibility to opioid dependence (Huang et al., 2016). Such findings indicate a possible relationship between AAS abuse and opioid abuse.

There is also data indicating the prominent participation of the glutamatergic neurotransmission in the reward circuitry. It has been largely recognized that glutamatergic neurotransmission in the NAc is associated with pain regulation (Ghalandari-Shamami et al., 2011). For instance, there is growing evidence that the antinociceptive response driven by cannabinoids within the basolateral amygdala is mediated by the NAc *N*-methyl-D-aspartate (NMDA) receptors (Ghalandari-Shamami et al., 2011). The involvement of the NAc glutamatergic signaling in the mechanisms of chronic neuropathic pain is also supported by the increase in the GluA1 subunit, without a concomitant rise in the GluA2 subunit levels of AMPA receptors both in the core and the shell of the NAc, found in an experimental model of neuropathic pain (Xu et al., 2015). In fact, AMPA represents the chief excitatory post-synaptic glutamate receptor. It is composed by four subunits (GluA1–4), which in turn are crucial to its function. Particularly in the NAc, GluA1, and GluA2 are the predominant subunits. The selective increase in the GluA1 subunit, results in the formation of GluA2-lacking AMPA receptors. Furthermore, the transmission through those receptors decrease the depressive symptoms related to pain (Goffer et al., 2013). Interestingly, the levels of GluA1 subunits was not altered in an experimental model of acute post-incisional pain. On the other hand, an increase in the levels of GluA1 with concurrent formation of GluA2-lacking AMPA receptors was observed in a model of persistent but reversible inflammatory pain as well as in a model of chronic neuropathic pain. Nevertheless, while GluA1 levels remained high in the chronic neuropathic pain model, they returned to the baseline levels as the pain resolved in the animal model of inflammatory pain (Su et al., 2015). In the same study, a significant decrease in the depressive symptoms associate with pain was obtained when blocking GluA2-lacking AMPA receptors in the persistent but not in the acute pain model. Therefore, it might be possible that the changes that occur in the GluA1 subunit of AMPA receptors in the NAc represent an adaptive mechanism to reduce depressive symptoms of chronic pain conditions (Su et al., 2015).

AMPAkines are drugs that enhance glutamate transmission (Lynch, 2006). They also have a high affinity for NAc AMPA receptors, which make such receptors excellent targets for those drugs, particularly to the purpose of pain control (Montgomery et al., 2009). As a matter of fact, it has been demonstrated that AMPAkines produce a relieve in both sensory and affective dimensions of persistent pain (Le et al., 2014). In addition, they improve post-operative pain, an effect mediated by NAc AMPA receptors (Su et al., 2016). According to the results of a previous study, the infusion of the AMPAkine CX546 directly into the NAC reduces sensory hypersensitivity and depression-like behaviors related to acute post-operative incisional pain as well as persistent post-operative pain, while the infusion of the highly specific AMPA receptor antagonist NBQX into the NAc, inhibits the antinociceptive effects of the CX546 (Su et al., 2016). More recently, the orexinergic system and particularly the orexin-2 receptors (OX2rs) located in the NAc, has also been raised as potential molecular targets for persistent inflammatory pain management (Yazdi et al., 2016).

Pain is an intricate multifaceted phenomenon, rather than only clinical symptom. Moreover, it requires especial attention, especially when evolving to a chronic state. Chronic pain afflicts a considerable proportion of the general population worldwide and can be considered one of the most challenging clinical conditions. Notwithstanding, the progress obtained in pain research throughout the last decades, many of its basic mechanisms are still obscure, including the development of chronic state from an acute condition. In this regard, the current scientific literature points to a pivotal participation of the reward/motivational circuitry in this process. Dissecting and unveiling the contribution of the reward circuitry to pain perception and modulation will enhance the understanding of chronic pain pathophysiology and perhaps allow the development of more efficient therapies for such debilitating conditions.

## Author Contributions

MFD, BSM, and AFD drafted, reviewed, and approved the manuscript.

## Conflict of Interest Statement

The authors declare that the research was conducted in the absence of any commercial or financial relationships that could be construed as a potential conflict of interest.
